# A genome-wide association study of quantitative computed tomographic emphysema in Korean populations

**DOI:** 10.1038/s41598-021-95887-7

**Published:** 2021-08-17

**Authors:** Sooim Sin, Hye-Mi Choi, Jiwon Lim, Jeeyoung Kim, So Hyeon Bak, Sun Shim Choi, Jinkyeong Park, Jin Hwa Lee, Yeon-Mok Oh, Mi Kyeong Lee, Brian D. Hobbs, Michael H. Cho, Edwin K. Silverman, Woo Jin Kim

**Affiliations:** 1 Department of Internal Medicine, School of Medicine, Kangwon National University, Chuncheon, Republic of Korea; 2 Division of Biomedical Convergence, College of Biomedical Science, and Institute of Bioscience & Biotechnology, Kangwon National University, Chuncheon, Republic of Korea; 3 Department of Radiology, Kangwon National University Hospital, Kangwon National University School of Medicine, Chuncheon, Republic of Korea; 4 Department of Internal Medicine, Dongguk University Ilsan Hospital, Goyang, Republic of Korea; 5 Division of Pulmonary and Critical Care Medicine, Department of Internal Medicine, College of Medicine, Ewha Womans University, Seoul, Korea; 6 Department of Pulmonary and Critical Care Medicine, Asan Medical Center, University of Ulsan College of Medicine, Seoul, Korea; 7 Epidemiology Branch, Division of Intramural Research, Department of Health and Human Services, National Institute of Environmental Health Sciences, National Institutes of Health, Research Triangle Park, NC USA; 8 Channing Division of Network Medicine, Brigham and Women’s Hospital and Harvard Medical School, Boston, MA USA; 9 Division of Pulmonary and Critical Care Medicine, Department of Medicine, Brigham and Women’s Hospital and Harvard Medical School, Boston, MA USA

**Keywords:** Genetics research, Chronic obstructive pulmonary disease

## Abstract

Emphysema is an important feature of chronic obstructive pulmonary disease (COPD). Genetic factors likely affect emphysema pathogenesis, but this question has predominantly been studied in those of European ancestry. In this study, we sought to determine genetic components of emphysema severity and characterize the potential function of the associated loci in Korean population. We performed a genome-wide association study (GWAS) on quantitative emphysema in subjects with or without COPD from two Korean COPD cohorts. We investigated the functional consequences of the loci using epigenetic annotation and gene expression data. We also compared our GWAS results with an epigenome-wide association study and previous differential gene expression analysis. In total, 548 subjects (476 [86.9%] male) including 514 COPD patients were evaluated. We identified one genome-wide significant SNP (*P* < 5.0 × 10^–8^), rs117084279, near *PIBF1*. We identified an additional 57 SNPs (*P* < 5.0 × 10^–6^) associated with emphysema in all subjects, and 106 SNPs (*P* < 5.0 × 10^–6^) in COPD patients. Of these candidate SNPs, 2 (rs12459249, rs11667314) near *CYP2A6* were expression quantitative trait loci in lung tissue and a SNP (rs11214944) near *NNMT* was an expression quantitative trait locus in whole blood. Of note, rs11214944 was in linkage disequilibrium with variants in enhancer histone marks in lung tissue. Several genes near additional SNPs were identified in our previous EWAS study with nominal level of significance. We identified a novel SNP associated with quantitative emphysema on CT. Including the novel SNP, several candidate SNPs in our study may provide clues to the genetic etiology of emphysema in Asian populations. Further research and validation of the loci will help determine the genetic factors for the development of emphysema.

## Introduction

Emphysema and chronic bronchitis are important features of chronic obstructive pulmonary disease (COPD), and recent studies show that imaging features are associated with adverse clinical outcomes of COPD^1^. Computed tomography (CT) evidence of emphysema is associated with worse prognosis even among subjects without airflow obstruction^2,3^. However, there is a lack of specific emphysema prevention and treatment techniques in part due to a limited understanding of pathobiological mechanisms.

Smoking is a major risk factor for emphysema, but emphysema is identified in non-smokers and the severity of emphysema among smokers varies greatly^4–6^. Emphysema has a genetic component with significant heritability and a previous study estimated its heritability to be approximately 25%^7,8^. Previous reports have described genetic determinants of emphysema and airway phenotypes in smokers with or without COPD^9^, emphysema in the general population^10^, distinct local histogram emphysema pattern^11^, and emphysema distribution^12^.

However, most emphysema genome-wide association studies (GWAS) were performed either exclusively or predominantly in European ancestry individuals. The Korean Obstructive Lung Disease (KOLD) and COPD in dusty areas (CODA) cohorts were constructed in South Korea collecting CT imaging and blood samples that enabled assessments of genetic associations with CT features. We investigated genetic determinants of emphysema severity in Korean cohorts and sought to relate these to gene expression and DNA methylation.

## Methods

### Study sample

Blood samples of 1056 subjects from the KOLD and CODA cohorts were genotyped. KOLD is a prospective cohort which recruited COPD patients from 16 university hospitals in South Korea. CODA is a prospective cohort conducted on subjects with airflow limitation and healthy volunteers living in dusty areas near cement plants in the Kangwon and Chungbuk provinces of South Korea. Details of the cohorts were described in previous papers^13,14^. Written informed consent was given by each participant. This study received ethical approval from the Kangwon National University Hospital IRB (KNUH 2012-06-007) and the Asan Medical Center IRB (Approval No. 2005-0345). This study was conformed to the tenets of the Declaration of Helsinki.

### Computed tomographic measurements

In the KOLD cohort, CT measurements were obtained using 16-channel multidetector row CT scanner (SOMATOM Sensation; Siemens Medical Systems, Erlangen, Germany). In the CODA cohort, CT measurements were obtained using dual source CT scanner (SOMATOM Definition, Siemens Healthcare, Forchheim, Germany). In both studies, all subjects were scanned at full inspiration in a supine position. Emphysema was calculated as percent of lung area below or equal to the − 950 HU threshold and log-transformed.

### Genotyping and quality control

**Figure 1:**
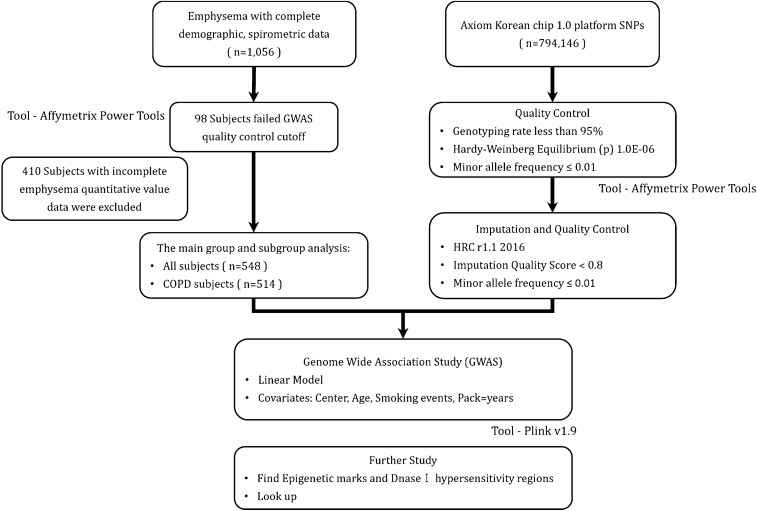
Study workflow.

We genotyped all subjects on Axiom KoreanChip 1.0 platform^15^. We removed low quality SNPs with low variant call rate, excessive heterozygosity and singletons, gender discrepancy, Hardy–Weinberg Equilibrium p < 0.001 and minor allele frequency < 0.01 using Affymetrix Power Tools and PLINK. After quality control, 586,966 SNPs with minor allele frequency of 1% or more were remained. (Fig. 1) Genotype imputation was performed at the Michigan Imputation Server using the HRC r1.1 reference panel. After imputation, SNPs with low imputation quality (R^2^ < 0.8) and minor allele frequency < 0.01 were removed. We combined original genotype and imputation data for analysis, and finally, 5,992,248 SNPs were analyzed for GWAS. The SNPs locations were based on National Center for Biotechnology Information (NCBI) Build 37.

### Genome-wide association analysis

We performed linear regression on natural log transformed emphysema index adjusted for age, sex, smoking status (never smoker, ex-smoker and current smoker), pack-years of smoking, and study center using PLINK (version 1.19)^16^. We also performed analyses only in COPD patients. We defined genome-wide significance as P < 5 × 10^–8^ and defined ‘candidate’ markers of interest at P < 5 × 10–6. Local association plots were generated around 600 kb in either direction of lead SNPs using LocusZoom in Asian genome (hg10/1000 Genome NOV 2014 ASN)^17^. Recombination rates were obtained using HapMap Phase II data^18^.

### Epigenetic marks and DNase I hypersensitivity regions in top SNPs

Potential functional information of SNPs which exhibit high linkage disequilibrium (LD) (r^2^ > 0.8, 1000 G Phase1, Asian population as reference) with the top associated SNPs was obtained using the HaploReg database (version 4.1) and the single-tissue expression quantitative trait loci (eQTL) data in the genotype-tissue expression (GTEx) consortium (lung and whole blood)^19,20^. The P-value threshold for significant eQTL was set at 5 × 10^–4^ in GTEx database.

#### Epigenome-wide association study (EWAS) related to emphysema

We performed EWAS of emphysema in blood DNA from 100 CODA subjects using the Infinium HumanMethylation450 platform. EWAS methods have been previously described in a study of emphysema index^21^.

### Comparison with previously published results

We performed look-ups of top SNPs in previously reported quantitative emphysema GWAS results which combined four study populations (COPDGene, ECLIPSE, National Emphysema Treatment Trial/Normative Aging Study, and GenKOLS)^9^. We also investigated whether the nearest genes of top SNPs were reported in previous differential expressed gene (DEG) analysis related to emphysema^22^.

## Results

### Baseline characteristics of study population

Baseline characteristics of study subjects are shown in Table 1. The mean age of all subjects was 71 years and 83.9% of all subjects were ever-smokers. Among a total of 548 subjects, 514 COPD patients who were defined as post-bronchodilator FEV_1_/FVC less than 0.7 and 34 subjects with normal spirometry had quantitative CT emphysema data (Fig. 1).

**Table 1:** Characteristics of subjects included in Genome-Wide Association Study.

	All (n = 548)	COPD (n = 514)
**Gender**		
Male, n (%)	476 (86.9)	456 (88.7)
Female, n (%)	72 (13.1)	58 (11.3)
Age, year	71.20 ± 7.60	71.12 ± 7.63
**Smoking**		
Never-smoker, n (%)	88 (16.1)	70 (13.6)
Ever-smoker, n (%)	460 (83.9)	444 (86.4)
Pack-years	30.95 ± 27.32	31.87 ± 27.30
**BMI (kg/m**^**2**^**)**	23.14 ± 3.25	23.13 ± 3.27
< 23.0, n (%)	262 (47.8)	243 (47.3)
23.0–24.9, n (%)	131 (23.9)	125 (24.3)
≥ 25.0, n (%)	155 (28.3)	146 (28.4)
Emphysema Index	12.46 ± 12.39	12.93 ± 12.49
FEV_1, % predicted	68.76 ± 22.70	66.54 ± 21.19

Data are mean ± SD for continuous variables and n (%) for categorical variables.

### GWAS in all subjects and COPD patients only

**Figure 2:**
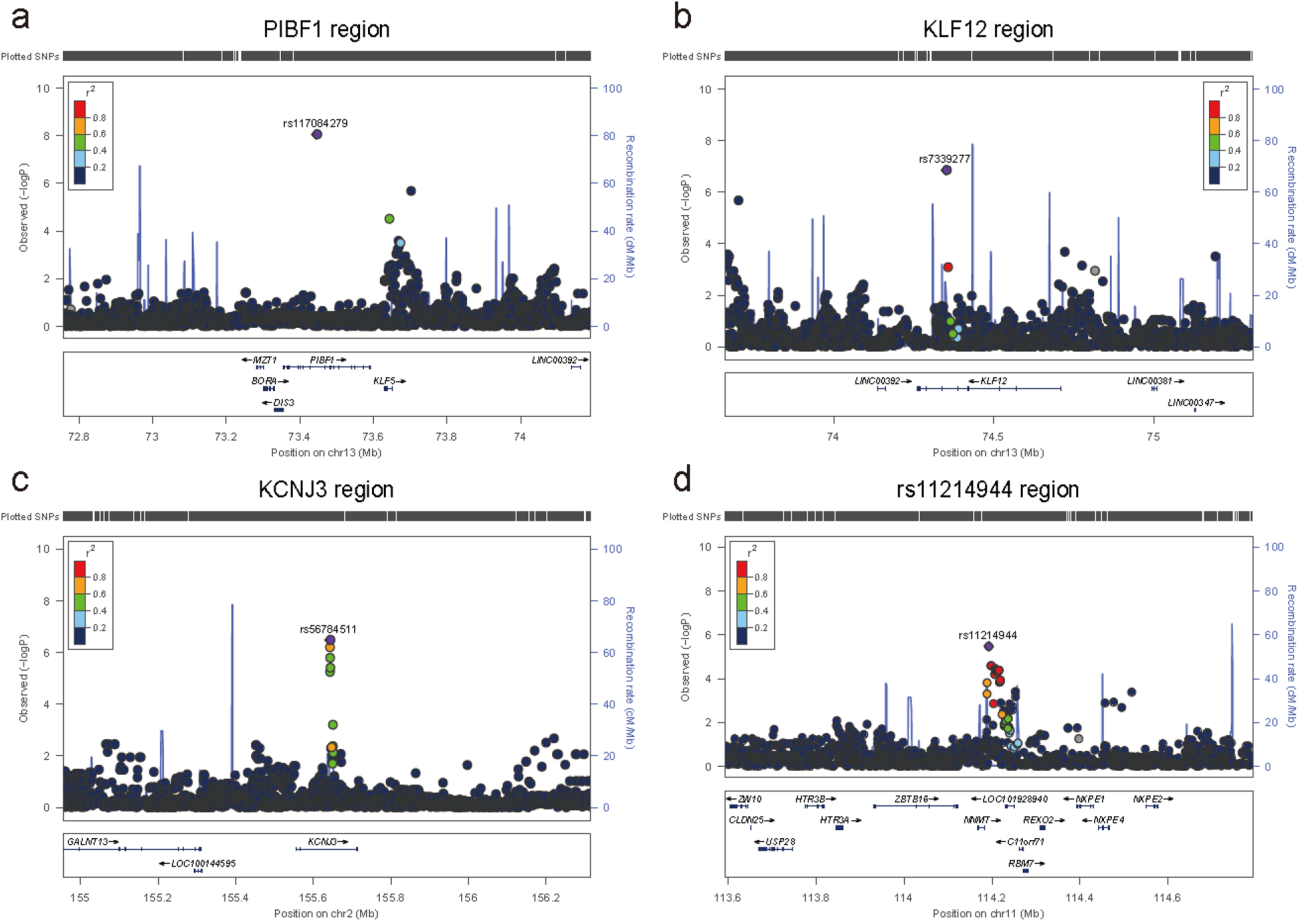
Regional plot for top SNPs in all subjects. Regional plot for ± 600 kb from top SNPs on PIBF1 (**a**), KLF12 (**b**), KCNJ3 (**c**) and rs11214944 (**d**) in total patient main model.

The Qq plot did not show systematic inflation in GWAS test statistics (lambda value 0.99 and 0.99 in all subjects and COPD patients, respectively (Supplementary Fig. S1). rs117084279 (MAF, 0.021), near the *PIBF1* gene was genome-wide significant in all subjects (Table 2, Fig. 2). Fifty seven SNPs in 19 loci identified in all subjects, and 106 SNPs in 16 loci in COPD patients, reached a pre-defined suggestive significance level (*P* < 5.0 × 10^–6^) forming the candidate SNPs (Table 2). Among the candidate SNPs, 24 SNPs overlapped in both groups. The top 20 SNPs of each group are shown in Tables 2 and 3.

**Table 2:** Top 20 SNPs of GWAS results in all subjects for emphysema.

SNP	Nearest gene	Genetic locus	Chr	Position (bp)	Ref/Alt	ALL main model				
						MAF	P value	β	SE	N
rs117084279	PIBF1	13q22	13	73,444,485	T/C	0.021	8.85E−09	− 1.37	0.235	548
rs7339277	KLF12	13q22	13	74,350,128	A/G	0.056	1.40E−07	− 0.76	0.143	545
rs142659816	–	20q13	20	48,635,162	G/A	0.037	3.11E−07	− 0.91	0.175	548
rs56784511	KCNJ3	2q24	2	155,641,769	A/G	0.067	3.29E−07	− 0.71	0.137	548
rs66771402	–	7p14	7	42,567,077	C/T	0.304	5.06E−07	0.36	0.071	548
rs67354756	KCNJ3	2q24	2	155,640,196	C/G	0.069	6.35E−07	− 0.68	0.135	548
rs1917213	–	7p14	7	42,577,872	A/T	0.310	6.36E−07	0.36	0.071	548
rs76470312	–	8p21	8	23,887,223	A/G	0.019	9.45E−07	− 1.20	0.241	548
rs143675498	CDH18	5p14	5	19,850,049	A/G	0.013	1.33E−06	− 1.42	0.291	547
rs6719455	KCNJ3	2q24	2	155,640,626	C/A	0.104	1.62E−06	− 0.55	0.113	547
rs6897483	–	5p13	5	29,735,073	A/T	0.192	1.67E−06	0.40	0.082	548
rs6897957	–	5p13	5	29,735,311	A/T	0.192	1.67E−06	0.40	0.082	548
rs10041866	–	5p13	5	29,738,133	C/A	0.193	2.06E−06	0.39	0.081	548
rs6880736	–	5p13	5	29,739,430	C/A	0.193	2.06E−06	0.39	0.081	548
rs7704866	–	5p13	5	29,736,738	A/G	0.193	2.06E−06	0.39	0.081	548
rs79879780	AL354720.1	13q22	13	73,699,403	C/T	0.026	2.10E−06	− 1.00	0.209	548
rs16950278	LOC101927284	13q32	13	95,533,306	A/G	0.015	2.18E−06	− 1.34	0.279	548
rs73547640	LOC101927284	13q32	13	95,531,311	T/C	0.015	2.18E−06	− 1.34	0.279	548
rs2168555	–	5p13	5	29,732,700	T/C	0.192	2.24E−06	0.39	0.082	548
rs149086677	FBLN2	3p25	3	13,629,075	G/A	0.028	2.30E−06	− 0.90	0.188	548

Model 1 was adjusted for Center, Age, Sex, Smoking event and Pack-years.

*ALL* All patients, *LNEI* natural logarithm of emphysema index.

*P value were less than 5.00E−06.

**Table 3:** Top 20 SNPs of GWAS results in COPD patients for emphysema.

SNP	Nearest gene	Genetic loci	Chr	Position (bp)	Ref/Alt	COPD main model				
						MAF	P value	β	SE	N
rs142659816	–	20q13	20	48,635,162	G/A	0.037	7.94E−08	− 0.94	0.172	514
rs6897483	–	5.p13	5	29,735,073	A/T	0.194	1.61E−07	0.43	0.081	514
rs6897957	–	5.p13	5	29,735,311	A/T	0.194	1.61E−07	0.43	0.081	514
rs2168555	–	5.p13	5	29,732,700	T/C	0.193	1.80E−07	0.43	0.081	514
rs6450715	–	5.p13	5	29,731,133	C/T	0.191	2.13E−07	0.43	0.081	514
rs7704866	–	5.p13	5	29,736,738	A/G	0.195	2.16E−07	0.42	0.081	514
rs16950278	LOC101927284	13q32	13	95,533,306	A/G	0.016	2.46E−07	− 1.40	0.267	514
rs73547640	LOC101927284	13q32	13	95,531,311	T/C	0.016	2.46E−07	− 1.40	0.267	514
rs7730624	–	5.p13	5	29,731,913	T/A	0.190	3.04E−07	0.42	0.081	514
rs1598675	–	5.p13	5	29,742,363	T/G	0.193	3.14E−07	0.42	0.081	514
rs6863232	–	5.p13	5	29,743,265	G/A	0.193	3.14E−07	0.42	0.081	514
rs11743334	–	5.p13	5	29,729,992	C/T	0.190	3.28E−07	0.42	0.081	514
rs6874707	–	5.p13	5	29,729,165	T/C	0.190	3.28E−07	0.42	0.081	514
rs10041866	–	5.p13	5	29,738,133	C/A	0.194	3.33E−07	0.42	0.081	514
rs6880736	–	5.p13	5	29,739,430	C/A	0.194	3.33E−07	0.42	0.081	514
rs6719455	KCNJ3	2q24	2	155,640,626	C/A	0.106	3.49E−07	− 0.57	0.111	513
rs7723786	–	5.p13	5	29,745,162	C/A	0.189	4.46E−07	0.42	0.082	514
rs67354756	KCNJ3	2q24	2	155,640,196	C/G	0.068	6.83E−07	− 0.67	0.133	514
rs2417661	–	9q31	9	108,681,246	A/G	0.012	6.97E−07	− 1.51	0.301	514
rs10117135	–	9q31	9	108,683,829	A/G	0.013	7.11E−07	− 1.45	0.289	514

Model 1 was adjusted for Center, Age, Sex, Smoking event and Pack-years.

*COPD* chronic obstructive pulmonary disease, *LNEI* natural logarithm of emphysema index.

*P value were less than 5.00E−06.

### eQTL results

We sought to determine whether candidate SNPs are located in regions that have effect on regulation of gene expression in lung and whole blood using the GTEx database. Three candidate SNPs in all subjects and 8 candidate SNPs in COPD patients were identified as eQTL in lung or whole blood in GTEx (Tables 4 and 5).

**Table 4:** eQTL among GWAS candidate SNPs in all subjects.

DB	Tissue	SNP Id	Gene symbol	P-value
GTEx	Lung	rs12459249	CTC-490E21.11	1.0.E−05
		rs11667314	CTC-490E21.11	6.3.E−06
	Whole blood	rs11214944	NNMT	9.9.E−05

*eQTL* expression quantitative trait loci.

**Table 5:** eQTL among GWAS candidate SNPs in COPD patients.

DB	Tissue	SNP Id	Gene symbol	P-value
GTEx	Lung	rs8070966	PROCA1	2.E−06
	Whole blood	rs62403725	KCTD20	6.E−07
		rs2242345	PROCA1	2.E−14
			ERAL1	6.E−07
		rs9892502	PROCA1	2.E−14
			ERAL1	6.E−07
		rs2874255	PROCA1	5.E−13
			ERAL1	8.E−07
			DHRS13	3.E−04
		rs968726	PROCA1	3.E−10
		rs9896239	PROCA1	3.E−11
		rs8070966	PROCA1	3.E−17
			ERAL1	2.E−07

*eQTL* expression quantitative trait loci.

### Comparing enhancer regions with GWAS results

To further explore the functional role of candidate SNPs, we examined our candidate SNPs and SNPs in LD with our candidate SNPs using the HaploReg database. We found 67 lead variants in candidate SNPs of all subjects and 69 lead variants in candidate SNPs of COPD patients were in LD (r^2^ > 0.8) with SNPs in promoter or enhancer histone marks or DNase I in lung tissue (Supplementary Table S1-A,B).

### Look-ups in DEG and EWAS data

**Figure 3:**
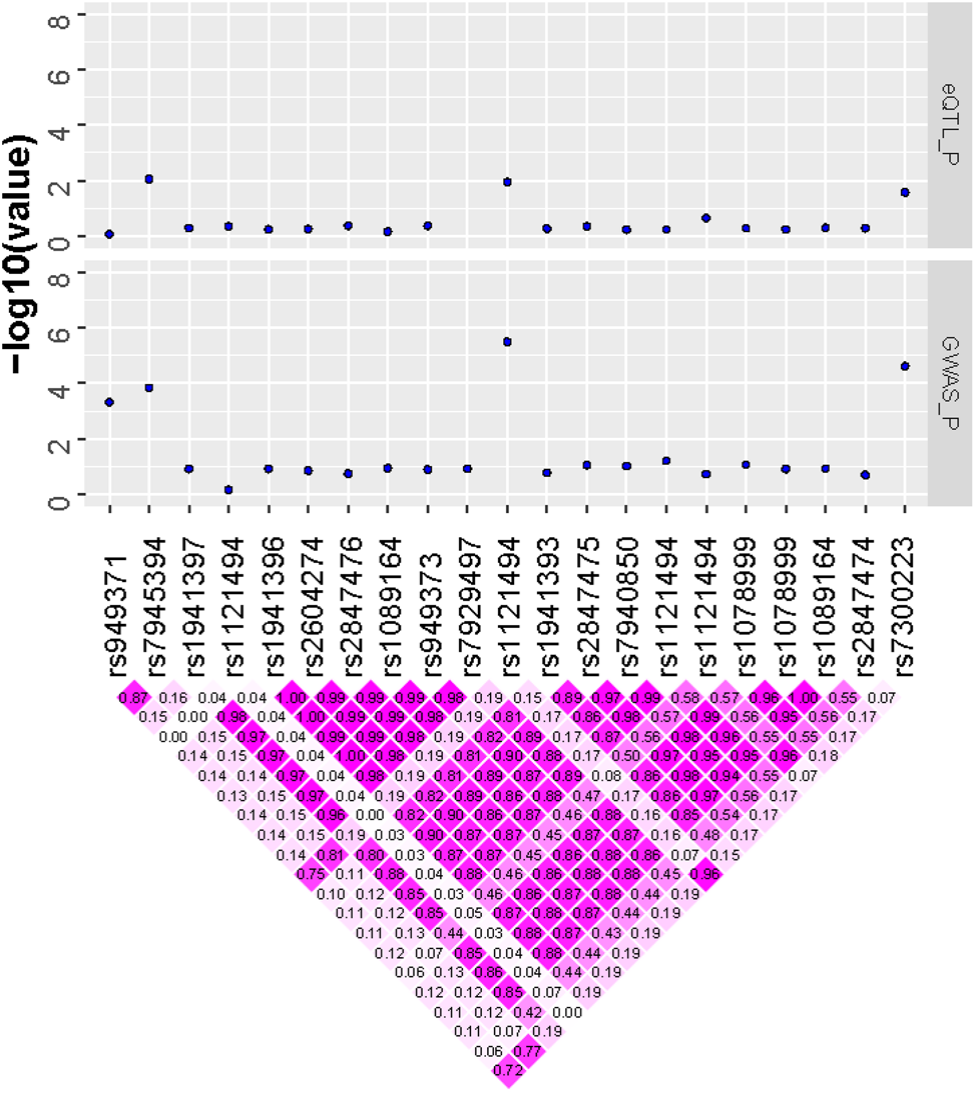
Integrating results of GWAS and functional studies.

Comparing with DEGs identified in a preceding emphysema RNA-seq analysis, *MACROD2* upregulation in emphysema overlapped with a region identified in the GWAS in all subjects. Similarly, *DOCK1, LARGE* and *ERAL1* also overlapped loci noted genes that is associated with SNPs identified in COPD patients (Table 6). In addition, several annotated genes of our candidate SNPs were also identified in our EWAS study related to emphysema with nominal level of significance (Supplementary Table S2). We plotted results of GWAS and functional studies together in Fig. 3.

**Table 6:** Differentially expressed genes in previous COPD transcriptome analysis.

Gene	Emphy exp	COPD exp	Non exp	ANOVA P value	SNP	P	Model
MACROD2	8.106	7.686	4.931	2.44.E−03	rs2423976	4.13E−06	All patients model
					rs4813189	3.68E−06	All patients model
DOCK1	13.813	13.903	11.072	3.57.E−07	rs6482989	7.24E−07	COPD patients model
ERAL1	12.589	12.588	13.952	3.22.E−03	rs2242345	1.37E−06	COPD patients model
					rs2874255	1.37E−06	COPD patients model
					rs9892502	1.37E−06	COPD patients model
LARGE	5.551	5.976	4.802	2.81.E−04	rs13056817	1.28E−06	COPD patients model
					rs1811103	1.28E−06	COPD patients model
					rs2018743	1.28E−06	COPD patients model
					rs2227023	2.43E−06	COPD patients model
					rs2413200	1.28E−06	COPD patients model
					rs2413201	1.28E−06	COPD patients model
					rs2899213	1.28E−06	COPD patients model
					rs379239	1.28E−06	COPD patients model
					rs412701	1.28E−06	COPD patients model
					rs419898	1.28E−06	COPD patients model
					rs444993	1.28E−06	COPD patients model
					rs447336	1.37E−06	COPD patients model
					rs454398	1.28E−06	COPD patients model
					rs4821187	2.43E−06	COPD patients model
					rs5749665	3.18E−06	COPD patients model
					rs5749668	1.28E−06	COPD patients model
					rs5749674	2.43E−06	COPD patients model
					rs5754676	1.28E−06	COPD patients model
					rs5754679	1.28E−06	COPD patients model
					rs5754682	1.28E−06	COPD patients model
					rs5754698	2.43E−06	COPD patients model
					rs5999109	1.28E−06	COPD patients model
					rs722659	1.28E−06	COPD patients model
					rs738942	1.28E−06	COPD patients model
					rs738950	1.28E−06	COPD patients model
					rs867124	1.28E−06	COPD patients model
MACROD2	8.106	7.686	4.931	2.44.E−03	rs2423976	4.13E−06	COPD patients model

### Look-ups in COPDGene data

In COPDGene emphysema results, we did not find any replicated SNP with our results (Supplementary Table S3).

## Discussion

In this study, we identified one genome-wide significant SNP in a novel candidate gene (*PIBF1*) and candidate SNPs (*P* < 5.0 × 10^–6^) for quantitative emphysema on CT in all subjects and COPD patients in Korean cohorts. We further found candidate SNPs (at a more liberal threshold of significance) were often eQTL in lung tissue, in linkage disequilibrium with variants in promotor or enhancer histone markers, or DNase I hypersensitivity sites in lung tissue or whole blood. Moreover, several SNPs were located near genes reported in preceding emphysema RNA-seq analysis or preceding EWAS studies.

In all subjects, we identified 2 candidate SNPs which were also identified as eQTL in lung tissue, near *CYP2A6. CYP2A6* has been associated with COPD and emphysema, and also smoking habits^23,24^. Genetic polymorphisms of *CYP2A6* result in altered activity of the CYP2A6 protein, affecting nicotine metabolism and smoking behavior^9,25,26^. This effect was also evaluated in the Asian population^27,28^. However, a relationship between this region and COPD and emphysema has not yet been described. Smoking is an important risk factor for the development of emphysema; therefore, it is possible that this variant contributes to the development of emphysema through smoking. Further research is needed to elucidate the causal relationship.

One of our candidate SNPs (rs11214944) near *NNMT* was identified as an eQTL and in LD with SNPs lying in enhancer histone marks in lung tissue. *NNMT* has also been identified as a gene differentially expressed according to severity of COPD^29,30^. Moreover, *NNMT* has been identified as differentially expressed in moderate emphysema compared to mild emphysema by more than sixfold^31^. Intriguingly, in a previous study, *NNMT* was one of differentially expressed genes in IL-6 signaling related to airway inflammation and remodeling^32^. Although the study was focused on airway inflammation, not emphysema, considering that IL-6 and its signaling play a main function in emphysema pathogenesis, we could expect association of *NNMT* and emphysema through IL-6^33^.

Although the MAF was very low, we identified one SNP, rs117084279, near *PIBF1*, that was genome-wide significant*.* Interestingly, *PIBF1* has also been identified as one component of centriolar satellite proteins and has an essential role in primary cilia formation and ciliary protein recruitment^34,35^. By whole genome siRNA-based functional genomics screen, mutation in *PIBF1* has been known to induce hereditary ciliopathy disease^36^. It has been well known that cigarette smoking causes structural and functional abnormality of cilia in bronchial epithelial cell^37–40^. Also, cigarette smoke is suggested to be responsible for genes associated with altered ciliary growth^41^. The relationship between ciliary function in airway epithelial cell and emphysema has not been well elucidated but our novel SNP could provide a potential link between them.

Likewise, *KLF12*and *KCNJ3* are genes near our candidate SNPs, which were also found to be associated with our EWAS results. Of them, *KCNJ3* was identified to associated with lung function and airway obstruction in previous studies, though there is no data on emphysema^24,42^. There is insufficient data to clarify that *KLF12* is related to emphysema in lung. However, previous studies suggested that *KLF12* gene, also known as the *AP-2rep* gene, functions as a transcriptional repressor of the *AP-2α* gene through a set of overlapping cis-regulatory promoter elements and a reciprocal regulation of both genes^43,44^. *AP-2α* is known to be involved in *ras* oncogene-mediated transformation and *myc*-mediated programmed apoptotic cell death^45,46^. Of interest, one study indicated that AP-2α protein was increased in lung of cigarette smoke exposure induced COPD rat model and this was also associated with increased cell apoptosis^47^. There are several mechanisms that likely contribute to the pathogenesis of emphysema^48–52^. One of them is apoptosis. Both animal COPD and human lung model suggest that apoptosis might be involved in the development of emphysema^53–55^. However, it has been unclear whether there is a direct relationship between *AP-2α* and apoptosis of cells in lung, results of our study yield novel insight of development and progression of emphysema and further experimental study is warranted.

In addition, we identified SNPs at a suggestive level of significance near *MACROD2* which is DEG identified in preceding emphysema RNA-seq analysis. *MACROD2* was associated with COPD and lung function in previous studies, but there is lack of data on the association with emphysema directly^56–58^. Although the reference study identified DEG according to presence of emphysema instead of quantitative value, integration with our GWAS result helps to identify meaningful genes among numerous genes^22^.

Although we did not find replicated SNPs in lookup results with COPDGene results, differential expression of genes and DNA methylation through high linkage disequilibrium with the genes could be suggested as a potential functional mechanism.

Inability to detect replicated SNPs in previous study is one of our limitations. This might be owing to small sample size, another limitation of our study, which can increase the false positive rates and decrease the statistical power. Even though a genome-wide significant SNP being identified, in view of both the relatively low MAF of the SNP and the small sample size of our study, further replication studies with larger population sizes are needed. Ethnic differences also could contribute to the inability of replication, and further Asian studies on emphysema are needed. Third, our study was not able to perform a meta-analysis with GWAS on emphysema or COPD in Asian population due to the lack of data. Comparing the results of meta-analysis with GWASs in different ethnicity would facilitate to elucidate the ethnic specificity. Fourth, the functional and biological impacts of the SNPs on emphysema are not identified in our study. Functional and integrated analysis may lead to a better understanding of pathophysiology of emphysema in Asian population. Also, we could not find out causal effects on emphysema of SNPs identified in our study. In further studies or meta-analysis including our study is needed to explore causal effects of the SNPs using Mendelian Randomization^59–63^. Finally, we had an interest in exploring whether the genetic cause could be a determining factor in emphysema regardless of smoking. Therefore, we focused on the results of the population that includes more never-smokers. However, owing to a small number of non-COPD subjects included in the total population, characteristics between the total and COPD populations may not be significantly different.

## Conclusions

In a genome-wide association study of emphysema in Korean COPD, we identified a new genome-wide significant association and several associations at suggestive significance. Ours is the first GWAS related to quantitative emphysema in Korean population. Further analysis of including replication in other independent cohorts and functional studies would yield insights into the development of emphysema. In particular, this work may be a starting point to investigate the aspects of the pathobiology of emphysema that are shared or unique across differing ancestries.

## Supplementary Information

Supplementary Figures.

Supplementary Tables.
