# Supplementary material for: A genome-wide association study of quantitative computed tomographic emphysema in Korean populations
Source: Sci Rep. 2021 Aug 17;11:16692. doi: 10.1038/s41598-021-95887-7 (PMC8371078; doi:10.1038/s41598-021-95887-7)

**A genome-wide association study of quantitative computed tomographic emphysema in Korean populations**

Sooim Sin^*1^, Hye-Mi Choi^*2^, Jiwon Lim^2^, Jeeyoung kim^1^, So Hyeon Bak^3^, Sun Shim Choi^2^, Jinkyeong Park^4^, Jin Hwa Lee, ^5^ Yeon-Mok Oh^6^, Mi Kyeong Lee^7^, Brian D. Hobbs^8,9^, Michael H Cho^8,9^, Edwin K Silverman^8,9^, Woo Jin Kim^1^

1 Department of Internal Medicine, School of Medicine, Kangwon National University, Chuncheon, Republic of Korea.

2 Division of Biomedical Convergence, College of Biomedical Science, and Institute of Bioscience & Biotechnology, Kangwon National University, Chuncheon, Republic of Korea.

3 Department of Radiology, Kangwon National University Hospital, Kangwon National University School of Medicine, Chuncheon, Republic of Korea.

4 Department of Internal Medicine, Dongguk University Ilsan Hospital, Goyang, Republic of Korea

5 Division of Pulmonary and Critical Care Medicine, Department of Internal Medicine, College of Medicine, Ewha Womans University, Seoul, Korea.

6 Department of Pulmonary and Critical Care Medicine, Asan Medical Center, University of Ulsan College of Medicine, Seoul, Korea

7 Epidemiology Branch, Division of Intramural Research, Department of Health and Human Services, National Institute of Environmental Health Sciences, National Institutes of Health, Research Triangle Park, NC, USA.

8 Channing Division of Network Medicine, Brigham and Women's Hospital and Harvard Medical School, Boston, MA, USA

9 Division of Pulmonary and Critical Care Medicine, Department of Medicine, Brigham and Women's Hospital and Harvard Medical School, Boston, Massachusetts, USA

*contributed equally

**Correspondence author:**

E-mail: [pulmo2@kangwon.ac.kr](mailto:pulmo2@kangwon.ac.kr) (WJK)

**Figure S1.** Quantile-quantile plots


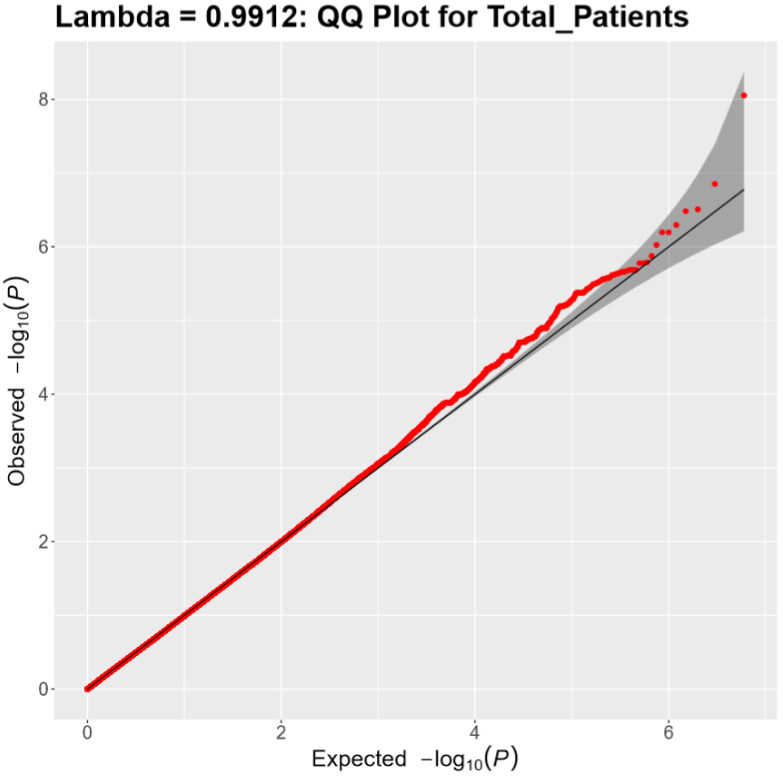

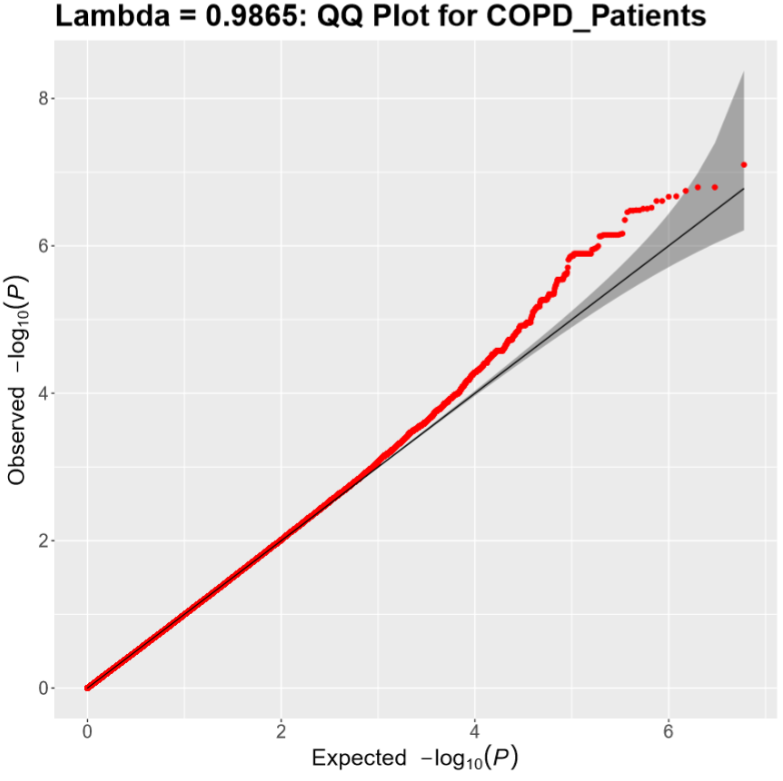


**Figure S2.** Manhattan plot for all subjects


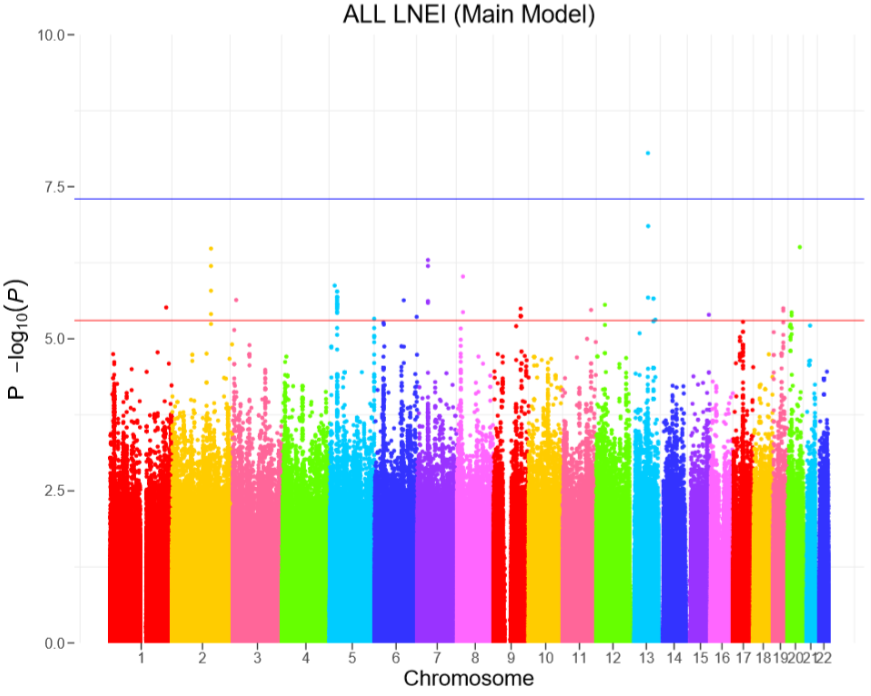


**Figure S3.** Manhattan plot for COPD patients


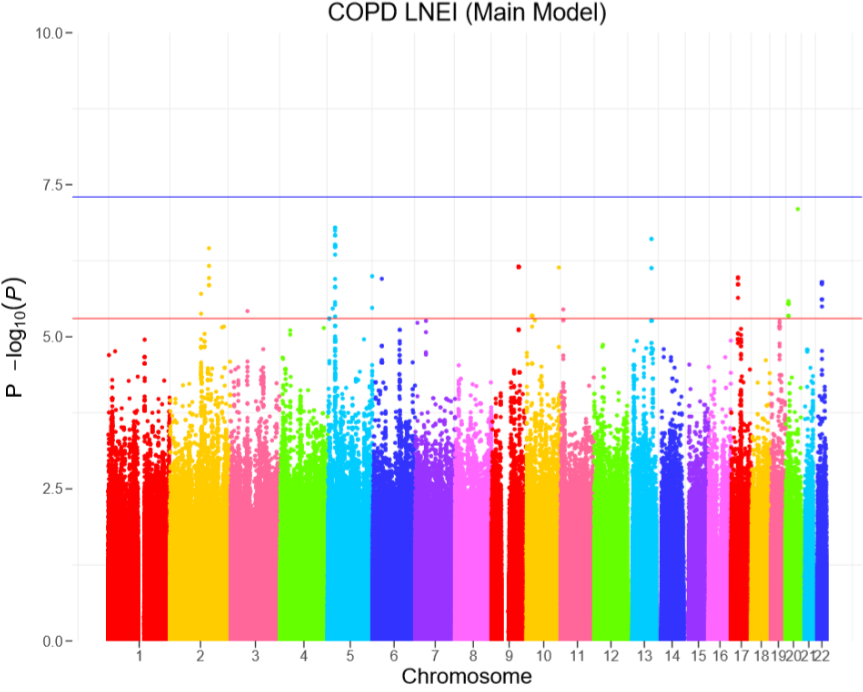

Supplement: Supplementary file 1 — Supplementary Figures. [file 41598_2021_95887_MOESM1_ESM.docx]
